# The impact of an Emergency Medical Technician basic course prior to medical school on medical students

**DOI:** 10.1080/10872981.2018.1474699

**Published:** 2018-05-28

**Authors:** Tasha R. Wyatt, Elena A. Wood, John McManus, Kevin Ma, Paul M. Wallach

**Affiliations:** a Educational Innovative Institute, Academic Affairs, Medical College of Georgia at Augusta University, Augusta, Georgia; b Department of Medicine, Academic Affairs, Medical College of Georgia at Augusta University, Augusta, Georgia; c Emergency Medicine Department, Medical College of Georgia at Augusta University, Augusta, Georgia; d Medical College of Georgia, Augusta University, Augusta, Georgia; e Department of Medicine, Academic Affairs, Medical College of Georgia at Augusta University, Augusta, Georgia

**Keywords:** Emergency Medical Technician, pre-matriculation, undergraduate medical education, medical students, qualitative analysis

## Abstract

**Background**: Previous research on Emergency Medical Technician (EMT) programs as an early clinical experience indicates that medical students’ confidence in patient care and team-building skills increases with participation. However, very little is known about the unplanned, long-term effects of EMT courses on medical students once they enter medical school.

**Objectives**: This study examined the immediate outcomes produced by the month-long summer EMT course and the unplanned outcomes that students reported 1 year later.

**Methods**: Pre/postsurveys were collected on all 25 students who graduated from the EMT course offered before their first year. These survey data were analyzed using a paired-samples *t* test. A subset of students (*N* = 14) consented to taking a survey and be interviewed on the lasting impact of their EMT experience. Interviews were conducted 10 months after the 2016 cohort completed the EMT course and at 22 months for the 2015 cohort. They were audio-recorded, transcribed, and analyzed using inductive content analysis.

**Results**: Survey results indicated that students’ confidence in patient care and team-building skills increased significantly for all identified skills at the *P* < 0.05 level. Overall confidence in patient care increased 1.5 points (*P* = 0.001) on 1–4 Likert-type scale. Overall confidence in team-building skills increased at 0.7 points (*P* = 0.01). Qualitative analysis of interviews discovered four themes, including the retention and transferability of practical skills, a developed understanding of team communication, comfort with patient interactions, and the development of a framework for assessing patients’ needs. Students applied the EMT skills in various extracurricular volunteering experiences and in clinical skills courses.

**Conclusions**: This study concludes that EMT programs have both immediate and lasting effects that seem to assist students with making sense of and navigating other learning opportunities. Specifically, EMT courses offered to students prior to their entry into medical school may help orient them to team-based health care and triaging patient care.

## Introduction

Medical education has increasingly provided students with early clinical experiences as a way to ease the transition from preclinical to clinical training, or prior to medical school [1–3]. Although many schools often grapple with which preclinical experiences yield the most effective results [1], previous research has demonstrated that Emergency Medical Technician (EMT) training increases medical students’ confidence in patient care and team-building skills [4]. To assess whether the pre-matriculation EMT training program would be a valuable addition at our medical school, evaluations from two cohorts of EMT students from the Medical College of Georgia (MCG) were assessed alongside data collected on participants in the form of semistructured interviews.

This short communication reports on our findings from this 2-year pilot study and builds on previous research on the value of EMT training as an early clinical experience. The addition of a qualitative interview was added above and beyond the data used in the previous study [4], because best practices in evaluation indicates researchers should include qualitative measures to explore unplanned effects [1]. The following sections provide a brief overview of our students’ pre-matriculation exposure at MCG, describe the design of the EMT course at our institution, and then offer data to support the value of a shortened version of EMT training as a preclinical experience.

## Methods

At our institution, first-year students have limited early clinical exposure unless they volunteered. Although several options have been considered to remedy this arrangement, at this point, MCG has not offered students such an opportunity as part of the curricular. However, as a way to expose students to the clinical environment, an EMT basic course was developed and offered to students as a pilot program during *pre-matriculation*. The 160-h course combined online and face-to-face lectures, small-group case studies, and procedural laboratory instruction, which facilitated graduates’ certification as licensed EMT providers. The course is limited to a select few in which only 20 slots are available to students each year. The students had the option to become EMT certified.

Participants in this study included 25 first- (*n* = 13) and second (*n* = 12)-year medical students who completed a month-long summer EMT course in 2014 or 2015. Data included pre/post- and lasting impact surveys, which have been described by Hofstra University’s researchers elsewhere [4], and were analyzed using a paired-samples *t* test. The pre/postsurvey asked scaled questions regarding students’ perceptions on the benefit of the EMT training on their patient care and team-building skills. On pre/postsurvey, questions ranged from ‘Not at all confident’ to ‘Very confident’ which used 1–4 scale [4]. On second survey, questions ranged on a scale from 1 to 5, with 1 representing ‘no impact’ and 5 representing ‘great impact’ for lasting impact [4]. In an attempt to build on earlier work on the value of EMT training, all students who completed the program during these 2 years were contacted and invited to participate in a ‘lasting impact’ survey and semistructured interview.

Out of the 25 students who completed the program in years 2015 and 2016, only a subset (*N* = 14) consented to the ‘lasting impact’ survey and interview. After being asked to complete a ‘lasting impact’ survey, they participated in a 30-minute semistructured interview exploring unplanned effects of the course. All interviews were conducted by two medical education researchers not affiliated with the EMT course. At the time of the study, interviews were conducted 10 months after the 2016 cohort completed the EMT course and 1 year and 10 months after the 2015 cohort completed the course. Interviews were audio-recorded, transcribed, and analyzed using inductive content analysis [5] until four themes emerged: Practical Skills, Team Communication, Patient Interactions, and a Framework for Assessing Patients’ Needs. Institutional Review Board (IRB) approved this study.

## Results

Pre/postsurvey results indicated confidence in patient care increased, which was statistically significant in 11 out of 13 items. Team-building skills showed a statistical significant increase from a baseline in all identified skills at the *P* < 0.05 level (Table 1). Overall confidence in patient care increased 1.5 points (*P* = 0.001) on 1–4 Likert-type scale. Overall confidence in team-building skills increased at 0.7 points (*P* = 0.01). Students rated a ‘lasting impact’ as having ‘some impact’ or ‘great impact’ on patient care (92% of students), and team-building skills (100%). Out of the 25 students, 22 became EMT certified.

**Table 1.** Students’ confidence in patient care (a) and team-building (b) skills.

	Paired differences		
a. Patient care skills	Mean	SD	*P* (two-tailed)
Approaching patients	0.5	0.71	0.05
Listening to patients	0.7	0.82	0.02*
Using appropriate body language	0.8	0.63	0.00*
Establishing rapport with patients	0.8	1.03	0.04*
Obtaining a medical history	1.4	0.84	0.00*
Using appropriate interview styles with patients	1.2	0.92	0.00*
Understanding a patient’s perspective	0.9	0.99	0.04*
Conducting a basic physical exam	1.2	1.13	0.00*
Responding to a patient’s medical issues	1.1	0.87	0.00*
Responding to a patient’s psychosocial issues	0.8	0.63	0.00*
Managing time in individual patient encounters	0.7	1.15	0.09
Identifying clinical dilemmas that require ethical decision making	1.1	1.28	0.02*
Overall conference in patient care skills	1.5	0.97	0.00*
	Paired differences		
b.Team-building skills	Mean	SD	*P* (two-tailed)
Understanding my role and responsibilities as a medical student	1.1	0.73	0.00*
Understanding the roles and responsibilities of others	1.0	0.81	0.00*
Developing a respectful working alliance	1.3	0.82	0.00*
Setting common goals	1.0	1.10	0.01*
Understanding the strengths of different team members	0.9	0.99	0.01*
Decision-making	1.0	0.66	0.00*
Adapting to a situation	1.0	0.66	0.00*
Being flexible	0.8	1.03	0.03*
Anticipating the needs of other team members	1.2	0.63	0.00*
Prioritizing work	0.8	0.78	0.01*
Conflict management	0.6	0.69	0.02*
Trusting other team members	0.7	0.94	0.04*
Overall conference in team-building skills	0.7	0.67	0.01*

*p < 0.05.

### Practical skills

Qualitative results indicated 50% of participants retained many of the practical skills taught in the course, which they transferred to their volunteering experiences. For example, one student described how he used his EMT skills while volunteering at the Salvation Army, putting him at an advantage over his peers, ‘EMT training trained us how to do vitals very well, and that’s one aspect a lot of other students struggle with’. An anesthesiology extern indicated his skills also extended into moments of crises. He expressed how this training helped him feel more comfortable knowing what to do in traumatic situations, such as when children come into the hospital with lodged objects in their throats. He explained, ‘I feel more comfortable than if I did not have the EMT background to handle some sort of traumatic thing’. Other students discussed the importance of transferring these practical skills to other courses. In these examples, students discussed the importance of knowing how to take blood pressures, splinting, and preventing shock, skills which they used in physical diagnosis (PD). As one student explained, ‘Having done a blood pressure before and taking a pulse, I already knew [how to do this] coming in to PD, whereas other students were [still] learning’.

### Team communication

Seventy-one percent of participants felt that they learned more about how much care goes on before a patient arrives at the hospital and how to communicate with other team members in an emergency situation. One student explained, ‘The EMT program definitely helped me see how you actually communicate during emergency situations’.

Beyond volunteering, students also applied what they learned in their problem-based learning (PBL) and PD courses. For example, one student described how the communication patterns in medical school differed from EMT training and the EMT experience helped her learn how to communicate better, ‘That’s something that’s unique about the EMT experience: coordinating with teammates on how to talk to each other and start treating a patient’. She indicated that this important skill can carry into her future practice as she works in team-based environments.

### Patient interactions

Fifty-seven percent of participants discussed the importance of early patient interaction and how this helped them feel more comfortable with standardized patients. Many of the students indicated that these early interactions were important because they did not have patient interaction skills prior to EMT training. For example, one student explained as follows:

Before the EMT course, I actually never worked with patients. I’d shadowed a physician and I worked with children during a teaching program, but I never worked [with a] patient … I was confident walking into [PD] because I already dealt with real patients.

Other students felt that early patient interaction on sensitive topics helped them navigate more difficult conversations later in PD. For example, one student described a conversation during his EMT training on a teenager’s sexual practices, which helped him navigate a later conversation on a similar topic. As he described, ‘I knew how to approach that conversation with tact, how to stay professional throughout that conversation [because it] was something I had already seen’. These early experiences provided models for students to rely on in medical school.

### Medical knowledge

Students indicated that the EMT course increased their medical knowledge in their ability to recognize whether patients were ‘sick’ vs. ‘not sick,' clinically triage, understand medical terminology, and implement first lines of action in acute situations. Forty-two percent indicated the EMT course provided them with frameworks for thinking about patient care. They indicated that the perspective they brought to their PBL and PD groups differed from the way their peers looked at cases. For example, several students expressed that most students lack preclinical experience and will order random tests when presented with a patient who is sick. However, those who completed the EMT training explained it helped them think about patients’ immediate needs before contemplating which tests to order. For example, one student said,

I usually … advance the group work, try to push the group in direction where the patients are stable enough that we can conduct the future tests that they desire. If the patient is not alive to perform these tests, then that’s not really a good course for the patient. I usually push for more of the [idea of] stabilize the patient and then we can talk about the different tests we can use.

Students who took the EMT course seem to have developed a framework for recognition that a patient required emergent care, initial evaluation, and management. One student described it as the ‘triage ability’. For him, EMT provided him with the following:

The ability to see someone and go “You need to go to the emergency room right now!”; or “You’re someone we need to keep an eye on for a little bit”; or “No you’re fine. This is something you can come back in a week for.” In clinical rotations, we get some of that, but it’s mostly the latter two.

This ability helps them think about patients’ needs in a way that the early years of medical school does not always provide.

## Conclusions

This study examined the immediate and lasting outcomes of an EMT course as a pre-matriculation experience for medical students. Results indicate that students’ increased confidence in working in teams and patient care persisted for over a year. Further, as students continued into medical school, other outcomes emerged, such as understanding how teams communicate, confidence in interacting with patients, and the development of a framework for thinking about immediate patients’ needs. Of note, this study’s major contribution is that the results suggest EMT training helps students develop frameworks for thinking about the immediate needs of a patient and the heuristic ‘Is the patient sick or not sick’ [6].

This study builds on earlier work that published on the value of EMT training as a preclinical experience [4]. It concludes that EMT programs may have lasting effects that assist students in navigating team-based environments where they interact with patients, and provide an additional framework for thinking about patient treatment. Compared to the previous study [4] in which students participated in mandatory EMT training integrated into first year of medical school for 9 weeks, on ‘lasting impact’ survey, our students rated their patient care skills at 92% compared to Hofstra’s students who rated it at 84%. Additionally, students rated their team-building skills at 100% compared to Hofstra’s students who rated it at 72%. We believe these findings are due to our students self-selecting to participate.

Future research should address this study’s limitations by expanding research to another institution, to have a comparison group, and to recruit a larger sample of students. Additionally, researchers might explore how EMT training is effective beyond students’ pre-clerkship years.
